# Oxidative Stress in Rats is Modulated by Seasonal Consumption of Sweet Cherries from Different Geographical Origins: Local vs. Non-Local

**DOI:** 10.3390/nu12092854

**Published:** 2020-09-18

**Authors:** Álvaro Cruz-Carrión, Ma. Josefina Ruiz de Azua, Miquel Mulero, Anna Arola-Arnal, Manuel Suárez

**Affiliations:** Nutrigenomics Research Group, Departament de Bioquímica i Biotecnologia, Universitat Rovira i Virgili, 43007 Tarragona, Spain; alvarojavier.cruz@urv.cat (Á.C.-C.); mariajosefina.ruiz@urv.cat (M.J.R.d.A.); miquel.mulero@urv.cat (M.M.); manuel.suarez@urv.cat (M.S.)

**Keywords:** cherries, polyphenols, antioxidant, phenolic signature, photoperiod, rhythms, seasons

## Abstract

Sweet cherries (*Prunus avium* L.) are a source of bioactive compounds, including phenolic compounds, which are antioxidants that contribute to protection against oxidative stress. It is known that the composition of cherries is influenced by external conditions, such as the geographic origin of cultivation, and that biological rhythms have a significant effect on oxidative stress. Therefore, in this study, Fischer 344 rats were exposed to various photoperiods and were supplemented with Brooks sweet cherries from two different geographical origins, local (LC) and non-local (NLC), to evaluate the interaction of supplementation and biological rhythms with regard to the oxidative stress status. The results indicate that the two fruits generated specific effects and that these effects were modulated by the photoperiod. Consumption of sweet cherries in-season, independently of their origin, may promote health by preventing oxidative stress, tending to: enhance antioxidant status, decrease alanine aminotransferase (ALT) and aspartate aminotransferase (AST) activities, reduce liver malondialdehyde (MDA) levels, and maintain constant serum MDA values and reactive oxygen species (ROS) generation.

## 1. Introduction

Many dietary phytochemicals, such as phenolic compounds, are synthesized as secondary metabolites that provide protection to plants against stresses [1]. Furthermore, these plant-derived chemicals consumed in relatively low doses activate adaptive cellular response signaling to produce stress resistance and other health advantages [2]. These molecules have been shown to be modulated by the environment, seasons, and various types of stress [3]. Hence, animals can use this signaling to anticipate seasonal changes and thus develop survival adaptations [4]. This phenomenon is known as xenohormesis and explains that bioactive compounds generated by plants under environmental stresses can provide resilience to stress and once consumed can generate survival benefits [2].

Humans routinely consume a wide range of phytochemicals from fruits, vegetables, and cereals. However, the daily consumption of fruits and vegetables differs widely around the world. Thus, a general pattern of increasing fruit and vegetable consumption in people with higher educational attainment is observed across all European Union Member States. In fact, in Spain, 77.4% of adults report eating fruits at least once a day [5]. Sweet cherry (*Prunus avium* L.) is one of the most widely consumed summer fruits across the temperate regions of Europe because of its good taste, health benefits, and comparatively low cost. Sweet cherries are seasonal and are available from May to August in Europe. However, in order to extend the natural harvest and marketing season for cherries, they are also imported from Turkey, Chile, Australia, or New Zealand [6].

Due to new eating styles and the relationship of fruit consumption with healthy effects and antioxidant capacity, cherries consumption has augmented [6]. Sweet cherries have been reported to be rich in anthocyanins, hydroxycinnamic acids, flavonols, and flavanols, which contribute to their health effects [6,7]. Polyphenols are secondary metabolites that have several activities. Thus, polyphenols from cherries manifest their health effects by various mechanisms, including antiproliferative and antioxidant effects [7]. However, phenolic composition is genotype-dependent and is influenced by climatic conditions, cultivars, harvesting season, and the environment (geographical origin) [8,9]. In fact, Serradilla et al. [9] reported that the cultivars Napoleona Grappolo and Sonata grown in southern Europe display a higher total phenolic content than the cultivars Lapins and Ferprime grown in northern Europe.

The antioxidant function of cherries is highly associated with their anthocyanin content, which inhibits lipoperoxidation more efficiently than traditional antioxidants [6]. An optimal antioxidant status protects against oxidative stress, which is defined as an imbalance between oxidants and antioxidants, causing damage to lipids, proteins, and cellular macromolecules. Oxidative stress is known to have a significant photoperiod-dependent effect [10]. The daily organization of behavior and physiology in animals is modulated by the circadian system, which functions as an endogenous clock and entrains the rhythms to the seasonal photoperiod changes [11]. These changes imply daily endogenous variations in the generation of free radicals, including reactive oxygen species (ROS) and other oxidants, as a consequence of the circadian rhythms of metabolism and behavior. The changes also involve exogenous daily fluctuations in the external environment, such as lighting and temperature [12]. Interestingly, daily rhythms of glutathione and malondialdehyde concentrations have been reported to be present in the brain [10]. Furthermore, it has been shown that transition dairy cows have higher erythrocyte levels of thiobarbituric acid-reactive substances (TBARS) during the summer [13]. There is evidence of fluctuations in cortisol content in plasma from goats depending on seasonal and ambient temperature [14].

To investigate this topic, we carried out a proof-of-concept study in which Fischer 344 rats were exposed to short, standard, and long photoperiods to simulate winter, autumn/spring, and summer light schedules, respectively, and the animals were supplemented with sweet cherries from two different growing locations. The main objective was to investigate the antioxidant status and the levels of oxidative stress biomarkers in rats consuming sweet cherries from different geographical origins in-season and out-of-season.

## 2. Materials and Methods

### 2.1. Chemicals and Reagents

Folin–Ciocalteu and *p*-dimethylaminocinnamaldehyde (DMACA) reagents were acquired from Fluka/Sigma-Aldrich (Madrid, Spain). Gallic acid, quercetin, (+)-catechin, Trolox, fluorescein, 2-thiobarbituric acid, trichloroacetic acid, reduced glutathione (GSH), monochlorobimane, glutathione S-transferase, and 2′,7′-dichlorofluorescein diacetate were purchased from Fluka/Sigma-Aldrich (Madrid, Spain). Cyanidin-3-*O*-rutinoside was purchased from PhytoLab (Vestenbergsgreuth, Germany). 2,2′-Azobis(2-methylpropionamidine) dihydrochloride (AAPH) was purchased from Acros Organics (Geel, Belgium).

The standard compounds to make up the calibration curve for the quantification of sweet cherry phenolics were prepared in acetone/water/acetic acid (70/29.5/0.5; *v*/*v*/*v*). Every three months, they were prepared again and stored protected from light at −20 °C.

### 2.2. Plant Fruit Material

Samples of sweet cherries (*Prunus avium* cv. Brooks) were used in this study. The Brooks sweet cherry was developed at the University of California-Davis and released to the public in 1987. This cultivar is a hybrid of Early Burlat and Rainier cherry, and it is characterized as a very high-quality, early-maturing sweet cherry that possesses the outstanding ability to develop large fruit [15]. These samples at commercial maturity were obtained from two different geographical origins: cherries from Tarragona, Spain (local sweet cherry, LC), were donated by the farmer in June 2018 (in-season consumption), and cherries from Cachapoal, Chile (non-local sweet cherry, NLC), were purchased in a local market in December 2018 (out-of-season consumption) (Supplementary Figure S1).

The edible part of the cherries was frozen in liquid nitrogen and ground. Then, the samples were freeze-dried for one week in a Telstar LyoQuest freeze-dryer (Thermo Fisher Scientific, Madrid, Spain) at −55 °C and ground (Moulinette 1, 2, 3, Moulinex); the powder was stored in amber flasks at room temperature until use.

### 2.3. Proximate Composition

To determine the sugar content, the soluble solid content in fresh fruit was measured in °Brix with a hand-held refractometer. In addition, the dietary components of local and non-local sweet cherry were characterized according to the official methods of analysis of the Association of Official Analytical Chemists (AOAC) [16]. The water content was measured as mass loss on heating (98 °C, 24 h). The ash content was calculated as the inorganic residue remaining after the water and organic matter were removed by heating (550 °C, 24 h). The protein was quantified by the Kjeldahl method (conversion factor 6.25), and the total lipid content was determined by continuous extraction with n-hexane in a Soxhlet extractor. The total dietary fiber (TDF) content was determined by treatment with heat-stable α-amylase, proteases from *Bacillus licheniformis*, and amyloglucosidase from *Aspergillus niger* (Sigma-Aldrich, Madrid, Spain) and by sequential weighing of the dry residue. The total carbohydrates content was calculated by subtracting the nutrient contents described above from the weight of LC and NLC. All assays were performed in triplicate using freeze-dried LC and NLC.

### 2.4. Extraction and Quantification of the Phenolic Compounds

The phenolic compounds in LC and NLC were extracted according to Iglesias-Carres et al. [17], where the extraction conditions were 80 mL/g, 65% methanol (1% formic acid), 72 °C, and 100 min under agitation of 500 rpm. The total contents of phenolics (TPC), anthocyanin (TAC), and flavanol (TFaC) in the LC and NLC extracts were analyzed by the methods described by Iglesias-Carres et al. [17]. Total flavonol content (TFoC) was measured according to the method described by Iglesias-Carres et al. [18].

### 2.5. Experimental Procedure in Rats

A total of 72 male Fischer 344 rats from 7 to 8 weeks of age were purchased from Charles River Laboratories (Barcelona, Spain) and randomly subdivided into 3 groups (*n* = 24, each) depending on the light exposure regimen. The groups were subjected to simulation of various photoperiods, including 6 h light/day (short photoperiod L6, which simulated the winter light schedule), 12 h light/day (standard photoperiod L12, which simulated the spring/autumn light schedule), or 18 h light/day (long photoperiod L18, which simulated the summer light schedule). Rats underwent photoperiod adaptation for 4 weeks. Thereafter, a daily supplementation period of 7 weeks with 100 mg LC dry weight (dw)/kg body weight (bw) [19], 100 mg NLC dw/kg bw, or vehicle (VH; glucose 21.2 mg/kg bw and fructose 21.2 mg/kg bw), in order to administer the same amount of sugars as those given to the cherry-supplemented rats, was implemented (*n* = 8, each). Supplementation was performed by voluntary oral administration between 8:00 a.m. and 9:00 a.m. (lights on at 7:00 a.m.) to avoid circadian interference. Throughout the experiment, the rats consumed a standard chow diet (AO4, Panlab, Barcelona, Spain) and tap water ad libitum. Rats were weighed every week, and their intake was monitored fortnightly. At the end of the experiment, the rats were deprived of food after supplementation with LC, NLC, or VH and were sacrificed by decapitation 1 h later. Blood was collected in non-heparinized tubes, incubated for 1 h at room temperature, and centrifuged (2000× *g*, 15 min, 4 °C) to obtain the serum. All collected organs and tissues were weighed and together with the serum were stored at −80 °C until use. The Ethical Committee of Animal Experimentation of the Rovira i Virgili University approved the experimental procedure (reference number 9495).

### 2.6. Biochemical Analysis

#### 2.6.1. Antioxidant Status

##### Oxygen Radical Absorbance Capacity

The method described by Huang et al. [20] was applied to estimate the oxygen radical absorbance capacity (ORAC). In brief, 25 µL of 73 mM AAPH was injected into each well from a 96-well microplate containing 25 µL of serum and 150 µL of 59.8 nM fluorescein. The fluorescence intensity of the samples was evaluated using a FLx800 multiple detection microplate reader (Biotek, Winooski, VT, USA) with excitation at 485 nm and emission measured at 528 nm every 2 min for 120 min. ORAC levels, which were calculated based on the standard curve of Trolox, are reported as mmol Trolox eq/L.

##### Reduced Glutathione Concentration

The hepatic levels of reduced glutathione (GSH) were assayed by the monochlorobimane fluorometric method [21]. Briefly, 10 µL of 100 µM monochlorobimane and glutathione S-transferase (1 U/mL) solution was added to 90 µL of homogenized liver; the homogenate was then incubated at room temperature for 30 min. GSH levels, which were determined based on the standard curve of GSH (Sigma-Aldrich), were measured using an FLx800 multi-detection microplate reader (Biotek) with excitation at 360 nm and emission measured at 528 nm and are expressed as µmol GSH eq/g liver.

#### 2.6.2. Oxidative Stress Biomarkers

##### Alanine Aminotransferase and Aspartate Aminotransferase Activity

Serum activities of alanine aminotransferase (ALT) and aspartate aminotransferase (AST) were assayed using an Eon BioTek spectrophotometer (Izasa, Barcelona, Spain) based on the protocols of commercial kits (Química Clínica Aplicada S.A., Amposta, Spain). The ALT and AST values are expressed as IU/L.

##### Malondialdehyde Production

Malondialdehyde (MDA) levels were determined by the TBARS assay [22]. Here, 20% trichloroacetic acid in 0.6 M HCl (1:1, *v*/*v*) was added to the serum, and the tubes were kept in ice for 20 min. Samples were centrifuged at 1500× *g* for 25 min, and TBA (120 mM in 260 mM Tris, pH 7) was added to the supernatant in a ratio of 1:5 (*v*/*v*); then, the mixture was boiled at 97 °C for 30 min. Spectrophotometric measurements at 540 nm were performed at 20 °C. The MDA values are expressed as µmol MDA/L.

The liver MDA levels were determined according to the method previously described for the serum assay. The MDA levels are shown as nmol MDA/g liver.

##### ROS Generation

ROS generation was quantified using the method described by Gabbia et al. [23] with some modifications. Briefly, 50 µL of liver homogenate was mixed with 500 µL of solution of 2′,7′-dichlorofluorescein diacetate (10 µM) diluted 1:200 in Tris-HCl buffer and the mixture was incubated for 40 min at 37 °C. The fluorescence intensity of the samples was assessed using an FLx800 multi-detection microplate reader (λex = 485 nm and λem = 528 nm). The ROS levels are expressed as FU/kg liver.

### 2.7. Statistical Analysis

The results of the fruit characterization are expressed as the mean ± standard deviation (SD). Student’s *t*-test was used to estimate significant differences (*p* < 0.05) between LC and NLC.

Biometric parameters and biomarker values (mean ± standard error of the mean, SEM) were subjected to a normality test and Levene’s test, then data were submitted to two- and one-way analysis of variance (ANOVA) in order to determine whether differences among the means exist, and then to the least significant difference (LSD) post hoc test comparison to allow determining which means differ, using Statistical Product and Service Solutions (SPSS) software (SPSS Inc., Chicago, IL, USA). Values of (*p* < 0.05) were considered statistically significant.

## 3. Results

In the present study, we evaluated whether consumption of sweet cherries from different geographical regions influences metabolism, antioxidant status, and oxidative stress in Fischer 344 rats when the cherries are consumed in-season or out-of-season. The rats were exposed to various photoperiods: a short photoperiod (cherry consumed out-of-season), a standard photoperiod (cherry consumed out-of-season), and a long photoperiod (L18) (cherry consumed in-season). In addition, sweet cherries were characterized based on their proximate and phenolic composition. The samples were obtained at commercial maturity, soluble solids content was measured as an indicator of maturity, LC showed 8.43 ± 0.45 °Brix, and NLC had 9.13 ± 0.12 °Brix. In addition, color intensity, that is the most commonly used indicator of maturity, was evaluated (Supplementary Figure S1), depending on the cultivar: red sweet cherries (e.g., Brooks) are commercially harvested when the skin color turns to solid bright red [6].

### 3.1. Proximate Composition of Local and Non-Local Brooks Sweet Cherry

The ash, protein, lipid, fiber, total carbohydrates, and sugar contents of LC and NLC were determined (Table 1). The ash values in both fruits were very similar; however, the remaining dietary components were statistically significantly different. In fact, LC had higher contents of protein, total lipids, and total dietary fiber that those in NLC. In contrast, NLC contained higher total carbohydrates and sugar contents. In this context, sweet cherries were dominated by carbohydrates (proportions higher than 79%), whereas the contents of ash, protein, and total lipids represented a small proportion of the LC and NLC proximate composition.

### 3.2. Phenolic Profiles of Local and Non-Local Brooks Sweet Cherry

The data of Table 2 indicate that the two fruits from different geographical origins used in the study (LC and NLC) had a specific phenolic signature. Indeed, the LC had higher contents of total phenolic compounds, total anthocyanin, and total flavanol than the NLC. On the other hand, the NLC had a higher total flavonol content. It should be emphasized that both LC and NLC had high contents of total anthocyanin, representing approximately 16% of TPC. Nevertheless, the total flavanol and total flavonol contents of both fruits were low, at under 0.62 mg/g dw.

### 3.3. Body Composition and Feeding Test

Table 3 presents the biometric parameters and feeding tests of rats exposed to the three photoperiods and administered with LC, NLC, or VH for seven weeks. All the groups underwent a similar weight evolution. In addition, no significant changes were identified in the majority of tissue weight and feeding tests after the administration of LC and NLC. Interestingly, regardless of the treatment received, L6 photoperiod-exposed rats exhibited a lower skeletal muscle weight than both L12 and L18 photoperiod-exposed rats. Similarly, mesenteric white adipose tissue (MWAT) weight was modulated by photoperiod: the rats exposed to L12 had a higher MWAT weight than the L6 and L18 rats. Moreover, photoperiod had considerable effects on body fat (%). Indeed, L6 photoperiod-exposed rats had lower body fat than the rats exposed to the L12 and L18 photoperiods.

### 3.4. Antioxidant Status

**Figure 1:**
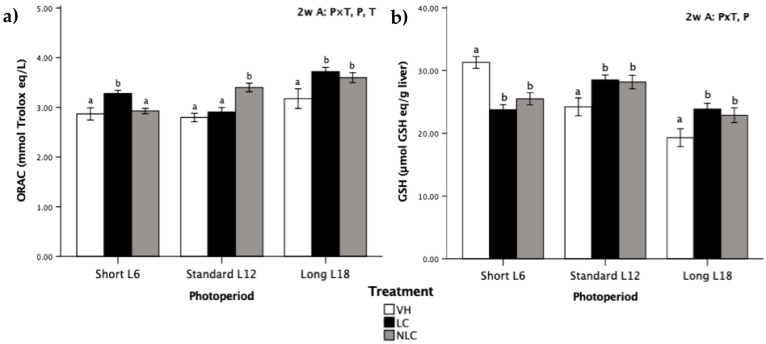
Levels of antioxidant status biomarkers in Fischer 344 rats exposed to three different photoperiods for 7 weeks and supplemented with local sweet cherry (LC), non-local sweet cherry (NLC), or vehicle (VH): (**a**) oxygen radical absorbance capacity (ORAC); (**b**) reduced glutathione (GSH) concentration. Data are expressed as the mean ± SEM (*n* = 8). Two-way ANOVA was performed to evaluate the differences between the groups, thus we could determine the effects of the photoperiod, P; the treatment, T; or the photoperiod × treatment interaction, P × T. Later on, one-way ANOVA was used to evaluate significant differences among the treatments (LC, NLC, and VH) within each photoperiod exposure (Short L6, Standard L12, and Long 18); these differences are indicated with different letters. Abbreviations: L6: 6 h light/day, L12: 12 h light/day, L18: 18 h light/day, ANOVA: analysis of variance.

The antioxidant status of Fischer 344 rats associated with LC and NLC consumption exposed to three different photoperiods is shown in Figure 1.

The antioxidant capacity was significantly affected by all the factors under evaluation (photoperiod, treatment, and photoperiod×treatment interaction) (Figure 1a). In this context, L18 photoperiod-exposed rats exhibited higher ORAC values than those exposed to L6 and L12 photoperiods. Moreover, rats that consumed cherries in-season (i.e., L18 sweet cherry-supplemented rats) had higher ORAC values than the control animals; the values increased by 16.98% by the administration of LC and by 13.21% in the case of NLC. On the other hand, LC administration in the L6 photoperiod and NLC administration in the L12 photoperiod also increased (by 14.29% and 21.86%, respectively) the levels of ORAC compared to those in other groups within the same photoperiod.

The data in Figure 1b indicate that the photoperiod induced significant differences in GSH concentration. Rats exposed to the long photoperiod had a lower GSH concentration than those exposed to short and standard photoperiods. Remarkably, both types of fruit had a similar impact on the pattern of GSH concentrations in all photoperiods; GSH levels were increased in rats exposed to the standard and long photoperiods (cherries consumed in-season) and decreased in rats exposed to the short photoperiod compared with the GSH levels in the VH rats.

### 3.5. Serum Oxidative Stress Biomarkers

**Figure 2:**
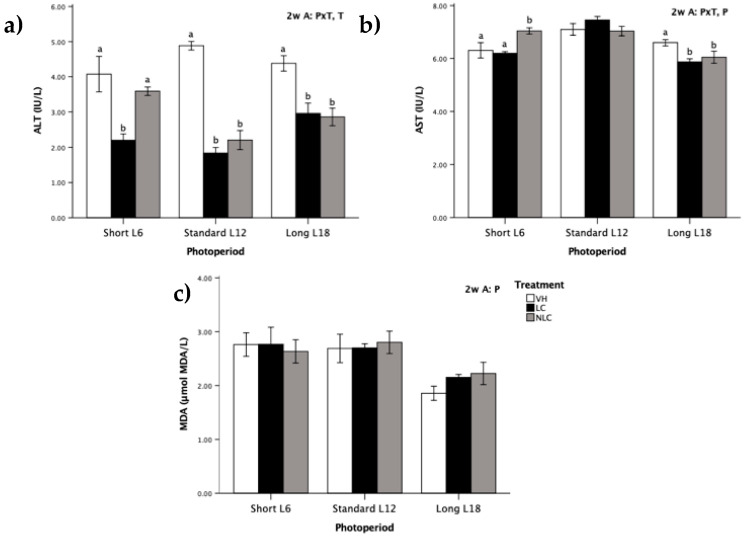
Serum oxidative stress biomarkers in Fischer 344 rats exposed to three photoperiods for 7 weeks and supplemented with local sweet cherry (LC), non-local sweet cherry (NLC), or vehicle (VH): (**a**) alanine aminotransferase (ALT) activity; (**b**) aspartate aminotransferase (AST) activity; (**c**) malondialdehyde (MDA) level. Data are expressed as the mean ± SEM (*n* = 8). Two-way ANOVA was used to evaluate the differences between the groups, thus we could determine the effects of the photoperiod, P; the treatment, T; or the photoperiod × treatment interaction, P × T. Later on, one-way ANOVA was used to evaluate significant differences among the treatments (LC, NLC, and VH) within each photoperiod exposure (Short L6, Standard L12, and Long 18); these differences are indicated with different letters. Abbreviations: L6: 6 h light/day, L12: 12 h light/day, L18: 18 h light/day, SEM: standard error of the mean, ANOVA: analysis of variance.

Each of those biomarkers was analyzed in the rat serum and was characterized by a specific and variable pattern (Figure 2). Regardless of the photoperiod exposition, consumption of sweet cherries diminished the ALT activity compared with that in VH supplementation (T effect, *p* ˂ 0.05, two-way ANOVA). This reduction was significant in all groups except NLC L6 (Figure 2a). The enzymatic activity of AST was significantly different between the photoperiods; it was highest in L12 rats, followed by L6 rats, and was the lowest in L18 rats (P effect, *p* ˂ 0.05, two-way ANOVA) (Figure 2b). Furthermore, both LC and NLC administration produced a similar decrease in AST activity compared with that in the control group in L18 photoperiod-exposed rats (cherries consumed in-season). On the other hand, in the opposite photoperiod (L6), fruit administration had different effects depending on the origin of the fruit. The NLC group had an increase in the enzymatic activity of AST compared with that in the other two groups.

No differences in the levels of serum MDA with regard to cherry administration were observed in any of the three photoperiods (Figure 2c). However, regardless of the treatment, L18 photoperiod-exposed rats had lower MDA levels than rats exposed to the short and standard photoperiods (P effect, *p* ˂ 0.05, two-way ANOVA).

### 3.6. Liver Oxidative Stress Biomarkers

**Figure 3:**
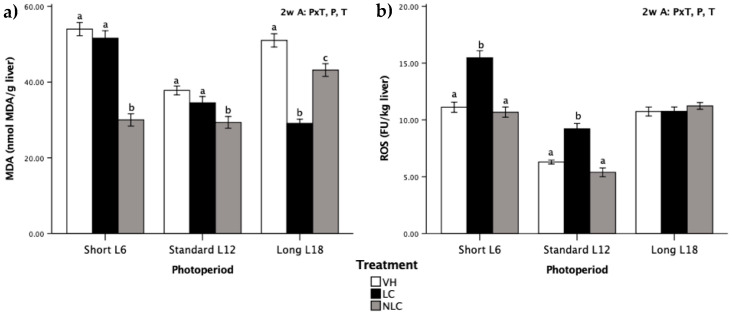
Liver oxidative stress biomarkers in Fischer 344 rats exposed to three different photoperiods for 7 weeks and supplemented with local sweet cherry (LC), non-local sweet cherry (NLC), or vehicle (VH): (**a**) malondialdehyde (MDA) level; (**b**) reactive oxygen species (ROS) generation. Data are expressed as the mean ± SEM (*n* = 8). Two-way ANOVA [3 × 3 factorial designs: treatment (LC, NLC, or VH) × photoperiod effect (L6, L12, or L18)] was used to evaluate the differences between the groups, thus we could determine the effects of the photoperiod, P; the treatment, T; or the photoperiod × treatment interaction, P × T. Later on, one-way ANOVA was used to evaluate significant differences among the treatments (LC, NLC, and VH) within each photoperiod exposure (Short L6, Standard L12, and Long 18); these differences are indicated with different letters. Abbreviations: L6: 6 h light/day, L12: 12 h light/day, L18: 18 h light/day, SEM: standard error of the mean, ANOVA: analysis of variance.

MDA levels and ROS generation were quantified in the liver (Figure 3). MDA and ROS were influenced by photoperiod, treatment, and P × T (*p* ˂ 0.05, two-way ANOVA), highlighting the impact of the performed treatments. Regardless of the photoperiod exposure, MDA levels were significantly different between the sweet cherry-supplemented rats (T effect, *p* ˂ 0.05, two-way ANOVA) (Figure 3a). Independent of the photoperiod, NLC administration produced a decrease in the MDA concentration compared with that in the VH group. On the other hand, LC supplementation reduced MDA levels compared with the levels of NLC and VH supplementation in L18 photoperiod-exposed rats (cherries consumed in-season). Interestingly, regardless of the treatment, L12 photoperiod-exposed rats had lower MDA and ROS values than rats exposed to both the L6 and L18 photoperiods.

ROS generation was influenced by photoperiod, treatment, and P × T (*p* ˂ 0.05, two-way ANOVA). The pattern was similar between the L6 and L12 photoperiod-exposed rats, highlighting that LC-supplemented rats had higher ROS generation than rats supplemented with NLC or VH (Figure 3b). On the other hand, LC and NLC had no effect on ROS generation in rats exposed to the L18 photoperiod.

## 4. Discussion

The literature indicates that circadian and circannual rhythms of living beings play an essential role in preventing excessive oxidative stress [12]. Thus, in our study, male Fischer 344 rats were chronically exposed for seven weeks to three photoperiods to mimic the day length of different seasons: L6 (winter season), L12 (autumn and spring seasons), and L18 (summer season), and the animal diets were supplemented with freeze-dried sweet cherries from two different growing locations, local fruit (LC) and non-local fruit (NLC), to investigate whether the season in which the fruit is consumed modulates the antioxidant status and oxidative stress in the animals and whether the geographical origin of sweet cherry cultivation can result in different effects.

The data indicate that the intake of LC and NLC produced differentiated physiological and metabolic responses depending on the photoperiod. Circannual rhythms had an influence on the antioxidant status of rats. In fact, L18 photoperiod-exposed rats had the lowest reduced glutathione (GSH) concentration (as shows the two-way ANOVA significance regarding P factor); interestingly, this group of animals had the highest ORAC values. Thus, 6 h light/day conditions apparently increased the GSH levels in the rats, whereas the 18 h light/day conditions decreased the GSH levels in the rats. These results are in agreement with the data of Escribano et al. [10], who determined that light reduced the GSH levels in the healthy animals and dark periods had the opposite effect. A similar result was reported in juvenile gibel carp (*Carassius auratus*), in which antioxidant-related metabolites (GSH) were decreased after long-term exposure to light [24]. Several studies reported a day–night rhythm in the hepatic GSH concentrations in rats with daily oscillating patterns [25]. In the case of the ORAC assay results, our study determined that the total antioxidant capacity increases in the animals exposed to the summer light schedule in agreement with the results obtained by Morera-Fumero et al. [26], who showed that ninety-eight healthy subjects had significantly higher total antioxidant capacity levels in the summer than in winter at three time points studied (09:00, 12:00, and 00:00 h).

Oxidative stress biomarkers can be classified as molecules that are modified by interactions with ROS in the microenvironment and molecules of the antioxidant system that change in response to increased redox stress [27]. Specifically, the quantification of ALT and AST can provide information about liver damage and the onset of oxidative stress hepatotoxicity. In addition, MDA produced during ROS-mediated peroxidation of polyunsaturated fatty acids is a widely used marker of oxidative stress [10]. In this study, oxidative stress biomarkers had a notable photoperiod-dependent effect, except ALT activity. Certainly, the photoperiod had an influence on serum MDA levels, which were diminishing concomitant to an increase in the daily light hours, and in the liver MDA levels, only LC rats followed this behavior. These results are in agreement with a study by Baydas et al. [28], who showed that MDA values were progressively augmented in dark periods. A rhythm of lipid peroxidation was also described in *Drosophila* males, and the peak of lipid peroxidation coincided with the evening maximum of locomotor activity [29]. On the other hand, AST activity at the hepatic level was modulated by photoperiod exposure, reaching the lowest activity when rats were exposed to the long photoperiod (two-way ANOVA Figure 2b). This finding is in agreement with the data reported by Hardeland et al. [30], who demonstrated that mouse liver AST activity is at its maximum during the dark photoperiod; our data also concur with the results reported by Çevik and Aslan [31], who observed that prolonged dark periods enhance the oxidation of nutritional fatty acids and that this oxidation triggers an increase in the AST levels. In contrast, the activity of ALT was not modulated by exposure to the photoperiods. This finding agrees with the data obtained by Mariné-Casadó et al. [4], who previously detected a lack of differences in the ALT values between rats exposed to three photoperiods; similar results confirmed that increasing light photoperiod had no significant effects [32]. It is important to define the status of these biomarkers because ALT and AST are intracellular enzymes released from damaged hepatocytes into the blood following hepatocellular injury [31].

In this study, sweet cherries were administered daily to the experimental rats at a dose of 100 mg freeze-dried fruit/kg body weight. Recalculation of this dose using dose translation from animal to human [33] to estimate the intake for a 60 kg human is equivalent to eating 5.12 g of fresh sweet cherries per day; this dose can contain a significant amount of nutrients and bioactive compounds that aid to achieve the daily intake recommended by the World Health Organization of at least 400 g of fruits and vegetables per day as part of a healthy eating pattern for the prevention of chronic diseases [34]. Eating fruits and vegetables is known to produce health benefits, and these effects are ascribed, at least in part, to their bioactive compounds; in particular, the sweet cherry fruit is a nutrient-dense food with a relatively low calorie content and significant amounts of important nutrients and bioactive food components such as fiber, polyphenols, carotenoids, vitamin C, and potassium, which have been reported to have beneficial health effects [7]. With regard to the overall impact of fruit consumption on the animals, overall, the intake of sweet cherries favored the antioxidant status in Fischer 344 rats because it significantly enhanced the antioxidant capacity (ORAC) by 13.66% and numerically increased the concentration of GSH. The antioxidant capacity of cherries has been evaluated by several methods, most commonly by ORAC [6]. In this study, ORAC was selected to evaluate the antioxidant capacity because of the antioxidant mechanism assessed by hydrogen atom transfer [35].

Cherry consumption promotes health, due to the high concentrations of bioactive compounds (such as polyphenols, melatonin, carotenoids, and vitamins E and C), which contribute to their antioxidant and anti-inflammatory effects. [7]. These results are related to the data of other studies, in which the intake of food stuffs rich in melatonin, such as cherries, grape juices, or beers, led to an increase in the plasma antioxidant capacity [36]; similarly, in another study, the short-term supplementation of Montmorency powdered tart cherries reduced immune and inflammatory stress and improved the maintained redox balance with a linear increase in the antioxidant activity at 24 and 48 h in aerobically trained individuals [37]. Similarly, cherry juice increased the total antioxidant status of the serum in recreational marathon runners by 10% over that detected in the placebo group, thus providing a broad spectrum of protection against inflammation and oxidative stress [38]. Sweet cherries are a significant source of various phenolic compounds that are associated with antioxidant activity due to their significant role in the stabilization of lipid peroxidation [7]. It has been attributed that one of the most important benefits of cherry consumption is the increase in the bioavailability of antioxidants [39]. Bioavailability of antioxidants contributes to preserving the appropriate redox balance after over-generation of ROS [37]. ROS produced during aerobic metabolism is known to cause oxidative damage to the macromolecules [27]. Living organisms are protected from ROS by several defence mechanisms, including antioxidant enzymes and low-molecular weight antioxidants, such as GSH [35]. GSH forms a part of the antioxidant defence systems produced by the body to protect the cellular constituents from the damages caused by ROS [35]. Furthermore, it is very important that fruit supplementation to animals in this study had important effects on ALT activity and MDA concentration in the liver resulting in a remarkable reduction in ALT enzymatic activity of 34.62% and in MDA values of 20.08%. The tendency to reduce the concentration of MDA in the liver of rats supplemented with LC and NLC confirmed the antioxidant effects of sweet cherries. Other authors studied the addition of sweet cherry fruit and leaves to a high fat-cholesterol diet and reported that the hepatic enzymatic activity and MDA values decreased in Wistar rats and that oxidative stress was reduced [40]. Indeed, our results indicate that eating sweet cherries can improve liver function and lipid peroxidation. This effect can result from the presence of bioactive compounds, especially phenolic acids, in *Prunus avium* L. The literature data suggested that hydroxycinnamic acids reduce the activities of hepatic enzymes [40].

Genetic and environmental factors can modulate the phenolic composition of fruit, and various phenolic profiles can be encountered in fruits of the same variety originating from different geographical locations. In detail, especially relevant factors include geographical origin of cultivation, environmental conditions (preharvest day and night temperature, light intensity), ripeness, and post-harvest conditions. The nutritional composition of the harvested fruit can be modulated by growing conditions [8]. In fact, Viljevac et al. [41] concluded that geographical location had a strong impact on polyphenol and anthocyanin contents of various sour cherry cultivars. However, current globalization makes it possible to obtain fruits and vegetables from various regions all over the world and all year round. Therefore, in this study, we evaluated two Brooks sweet cherry cultivars from different geographical regions administered for seven weeks. The proximate composition and phenolic profile of LC and NLC presented a different nutritional composition and a specific phenolic signature. The results indicate that the levels of TAC, which is the main group of phenolic compounds in sweet cherries [6,7,17], were significantly different between LC and NLC, demonstrating the environmental influence on anthocyanin synthesis. These differences in TAC are in agreement with the data on bilberry grown at various altitudes in the eastern Alps of Austria, where anthocyanin levels were lower at higher altitudes [42]. On the other hand, the notably higher content of TFoC in NLC can be caused by the temperature at the growing location, because the growing temperature in December 2018 of Chile was higher in comparison with Spain in Jun 2018 [43]. This finding is in agreement with the results found by Josuttis et al. [44], who in strawberries observed that higher temperatures increased the content of total flavonols. In the same way, TFaC was different between the two fruits, being higher in LC than NLC. We were unable to access the specific conditions under which the non-local cherries were harvested and this could have an impact on the obtained results. Moreover, each fruit generated different effects on the antioxidant capacity and biomarkers of oxidative stress monitored in the rats supplemented with the fruits. Thus, regardless of photoperiod exposure, LC and NLC administration had statistically similar effects on the antioxidant status levels in rats; however, LC-supplemented rats had higher numerical values. This effect might be related to the fact that LC contained higher levels of fiber and phenolic compounds compared with that in NLC; similar differences were reported to improve the antioxidant status [45]. Similarly, ALT activity was remarkably decreased by LC supplementation. In contrast, NLC supplementation diminished the ROS values, and this effect may be a consequence of the antioxidant activity that scavenges ROS generated in the plasma. These biological activities of LC and NLC can be related to the individual and specific nutritional composition and phenolic signature of each fruit type.

According to xenohormesis theory, animals are able to adapt their physiology to the changes in the environmental conditions by consuming phytochemicals, chemical signals synthetized by plants. Consequently, the beneficial effects provided by sweet cherries consumed in-season are different in the other two photoperiods with simulated out-of-season consumption; overall, the metabolic response generated by eating fruit with a seasonally distinctive phenotype depends on the circannual rhythms, and this response can be altered or distorted when the fruit is consumed out-of-season. [11,46]. When LC and NLC are ingested in-season (L18, summer season), a notable increase in the antioxidant status of the animals housed under these conditions was observed. Another remarkable effect of the in-season consumption of sweet cherries was manifested as a reduction in the enzymatic activity of both ALT and AST. Moreover, L18 photoperiod-exposed rats experienced a substantial lowering of the MDA levels in the liver. It is important to emphasize that the in-season intake of cherry maintained constant levels of circulating serum MDA and ROS generation. Otherwise, out-of-season fruit eating causes an alteration in the characteristic seasonal metabolism [46]. Indeed, in this study, when the sweet cherries were consumed out-of-season, a markedly different pattern of antioxidant and oxidative stress biomarker values was observed. In fact, eating sweet cherries during the winter season lowered the GSH levels, which is opposite to the effect of in-season consumption. Moreover, the intake of NLC in L6 tends to increase the enzymatic activity of AST, compared with both LC and VH groups, while the consumption of the fruit out-of-season (winter, spring, or autumn) influenced the ROS levels: NLC consumption produced a tendency similar to that observed in the control group and LC intake increased the generation of ROS. Similarly, recent studies of our group have found that eating sweet cherries out-of-season may induce erroneous metabolic signaling in dietary-induced obese F344 rats, such as increases in whole-body fat oxidation and circulating levels of glucose and insulin [46]; changing the morphology of white adipose tissue, enhancing the cell area, and reducing the amount of adipocytes [47]; and, eventually, modulating the hypothalamic leptin system regulating *Agrp* and *Ptp1B* mRNA levels [48].

## 5. Conclusions

In summary, this study demonstrates that in-season consumption of sweet cherries can promote health by preventing an increase in oxidative stress. Sweet cherries enhance the antioxidant status, decrease the enzymatic activity of ALT and AST, reduce the MDA levels in the liver, and maintain constant serum MDA levels and ROS generation. Otherwise, out-of-season consumption of the fruit can induce erroneous signaling. In terms of geographical origin, local and non-local Brooks sweet cherries had a different nutritional composition and a specific phenolic signature, thus generating differentiated and particular effects on the antioxidant status and oxidative stress in Fischer 344 rats when consumed in-season, with significant emphasis on reducing the liver MDA levels by supplementation with LC. Our results emphasize the significance of the consumption of local and seasonal fruits to achieve ideal health. However, further studies with different local and non-local cherries are needed to confirm these findings.

## Figures and Tables

**Table 1:** Proximate composition of local sweet cherry (LC) and non-local sweet cherry (NLC).

Nutrients	LC	NLC	*p* Value
Ash	2.05 ± 0.15	2.11 ± 0.35	0.81
Protein	6.41 ± 0.11	5.51± 0.07	0.01
Total lipids (fat)	1.01 ± 0.01	0.70 ± 0.07	0.03
Fiber, total dietary	10.95 ± 0.03	10.74 ± 0.06	0.04
Total carbohydrates, by difference	79.50 ± 0.18	80.78 ± 0.34	0.04
Sugars, total	43.30 ± 2.31	50.08 ± 0.68	0.02

The results are expressed as g/100 g dw ± SD (*n* = 3). SD: standard deviation.

**Table 2:** Phenolic profiles of local sweet cherry (LC) and non-local sweet cherry (NLC).

Phenolic Compounds	LC	NLC	*p* Value
TPC (mg GA eq/g dw)	8.17 ± 0.20	7.64 ± 0.41	0.03
TAC (mg Cy3R eq/g dw)	1.31 ± 0.02	1.23 ± 0.03	0.02
TFaC (mg Cat eq/g dw)	0.44 ± 0.02	0.38 ± 0.03	0.02
TFoC (mg Quer eq/g dw)	0.55 ± 0.00	0.63 ± 0.05	0.04

The results are expressed as mg of phenolic components per gram of dry weight (mg/g dw) ± SD (*n* = 3). Abbreviations: TPC: total phenolic content, TAC: total anthocyanin content, TFaC: total flavanol content, TFoC: total flavonol content, GA: gallic acid, Cy3R: cyanidin-3-O-rutinoside, Cat: catechin, Quer: quercetin, SD: standard deviation.

**Table 3:** Biometric parameters and feeding tests of Fischer 344 rats exposed to three different photoperiods for 7 weeks and supplemented with local sweet cherry (LC), non-local sweet cherry (NLC), or vehicle (VH).

	Short L6			Standard L12			Long L18			2wA
	LC	NLC	VH	LC	NLC	VH	LC	NLC	VH	
**Biometric parameters**										
Body weight gain (g)	57.1 ± 5.14	52.9 ± 3.15	44.6 ± 4.12	55.3 ± 1.24	52.9 ± 3.30	54.0 ± 3.50	50.0 ± 1.55	53.5 ± 4.00	51.5 ± 0.72	n.s.
Skeletal muscle (g)	3.64 ± 0.28	3.92 ± 0.16	4.13 ± 0.10	4.40 ± 0.07	4.18 ± 0.09	4.30 ± 0.10	4.24 ± 0.12	4.33 ± 0.15	4.43 ± 0.07	P
BAT (g)	0.45 ± 0.03	0.45 ± 0.05	0.40 ± 0.06	0.47 ± 0.03	0.39 ± 0.01	0.39 ± 0.03	0.34 ± 0.03	0.36 ± 0.05	0.44 ± 0.03	n.s.
EWAT (g)	8.61 ± 0.23	8.73 ± 0.45	9.01 ± 0.44	10.10 ± 0.93	8.94 ± 0.50	9.49 ± 0.47	9.20 ± 0.58	9.21 ± 0.57	9.88 ± 0.74	n.s.
IWAT (g)	2.87 ± 0.36	2.46 ± 0.21	2.83 ± 0.37	2.80 ± 0.21	2.50 ± 0.27	2.78 ± 0.19	2.52 ± 0.27	2.42 ± 0.22 *	3.35 ± 0.37	n.s.
MWAT (g)	4.69 ± 0.26	4.64 ± 0.27	5.56 ± 0.50	6.05 ± 0.36	5.22 ± 0.38	5.52 ± 0.30	5.39 ± 0.37	4.51 ± 0.24	5.12 ± 0.30	P
Body fat (%)	4.30 ± 0.19	4.43 ± 0.11	4.37 ± 0.16	4.68 ± 0.29	4.71 ± 0.20	4.78 ± 0.20	4.74 ± 0.23	5.11 ± 0.31	4.62 ± 0.16	P × T, P
**Feeding tests**										
Food intake (kcal/day)	56.9 ± 1.23	55.1 ± 1.62	54.1 ± 0.29	56.2 ± 1.13	53.8 ± 1.06	55.4 ± 1.15	54.0 ± 0.40	55.1 ± 1.00	54.9 ± 0.47	n.s.
FE (g/100 kcal)	2.76 ± 0.16 *	2.64 ± 0.14 *	2.16 ± 0.14	2.50 ± 0.04	2.54 ± 0.14	2.46 ± 0.18	2.64 ± 0.11	2.89 ± 0.16	2.75 ± 0.20	n.s.

Data are expressed as the mean ± SEM (*n* = 8). Body weight gain (g) was determined as the difference between final rat weight and weight at the beginning of the treatment. The skeletal muscle weight represents the total weight of the soleus and gastrocnemius muscles. Food efficiency (FE) was calculated as body weight gain per kcal consumed over the entire treatment and is expressed as g/100 kcal. * *p* ˂ 0.05 vs. VH in the photoperiod; two-way ANOVA was used to evaluate the differences between the groups. P, photoperiod effect; T, treatment effect; P × T, photoperiod × treatment interaction effect. Abbreviations: L6: 6 h light/day, L12: 12 h light/day, L18: 18 h light/day, n.s.: not significant, BAT: brown adipose tissue, WAT: white adipose tissue, EWAT: epididymal WAT, IWAT: inguinal WAT, MWAT: mesenteric WAT, SEM: standard error of the mean, ANOVA: analysis of variance.

## Supplementary Materials

The following are available online at https://www.mdpi.com/2072-6643/12/9/2854/s1, Figure S1: Representative images of Brooks sweet cherries from two different geographical origins: (**a**) local sweet cherry (LC) from Tarragona, Spain; (**b**) non-local sweet cherry (NLC) from Cachapoal, Chile.
