# Supplementary material for: Oxidative Stress in Rats is Modulated by Seasonal Consumption of Sweet Cherries from Different Geographical Origins: Local vs. Non-Local
Source: Nutrients. 2020 Sep 18;12(9):2854. doi: 10.3390/nu12092854 (PMC7551698; doi:10.3390/nu12092854)
Supplement: Supplementary file 1 [file nutrients-12-02854-s001.pdf]

Supplementary Materials: The following are available online at www.mdpi.com/xxx/s1, Figure S1: Representative images of Brooks sweet cherries from two different geographical origins: (a) Local sweet cherry (LC) from Tarragona, Spain; (b) Non-local sweet cherry (NLC) from Cachapoal, Chile.

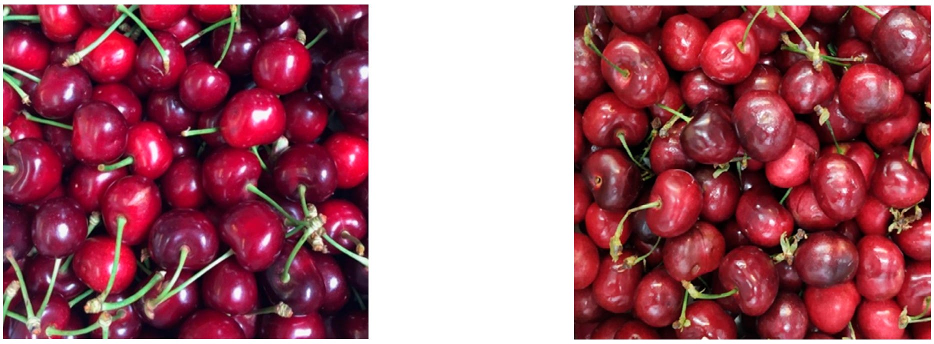

(**a**) (**b**)

Figure S1. Representative images of Brooks sweet cherries from two different geographical origins: (**a**) Local sweet cherry (LC) from Tarragona, Spain; (**b**) Non-local sweet cherry (NLC) from Cachapoal, Chile.
